# Synthesis and Characterization of Leucite Using a Diatomite Precursor

**DOI:** 10.1038/s41598-019-46569-y

**Published:** 2019-07-11

**Authors:** Daniela Novembre, Domingo Gimeno, Brent Poe

**Affiliations:** 1 Dipartimento di Ingegneria e Geologia, Università degli Studi “G.D’Annunzio”, Via dei Vestini 30, 66013 Chieti, Italy; 2 Department Mineralogia, Petrologia i Geologia Aplicada, Universitat de Barcelona, 08028 Barcelona, Spain

**Keywords:** Planetary science, Solid Earth sciences

## Abstract

Leucite is nowadays an important component in ceramic restoration systems with particular suitability to dental porcelains. The leucite synthesis from a hydrothermally-derived precursor is here presented. A silicate solution was prepared by mixing a naturally derived amorphous silica (diatomitic rock from Crotone, southern Italy) with potassium hydroxide and an aluminate solution was obtained by mixing aluminium hydroxide and potassium hydroxide. Three mixtures of varying ratios of aluminate and silicate solutions were prepared and submitted to hydrothermal treatment at 150 °C for one hour. Subsequently these hydrothermal precursors were subjected to calcination at the temperature of 1000 °C for variable time intervals, thus resulting in 3 series of syntheses. The synthesis run 3 turned out to be the best from the point of view of temporal yield showing the crystallization of the leucite after only 15 hours of heat treatment. The products of synthesis run 3 were fully characterised by Powder X-Ray Diffraction, Inductively Coupled Plasma Optical Emission Spectrometry, Infrared Spectroscopy and Thermal Analysis. The amorphous phase in the synthesis powders was estimated by quantitative phase analysis using the combined Rietveld and reference intensity ratio methods. Density of leucite was also achieved by He-pycnometry. The use of a cost effective starting material such as a diatomite in the experimental route makes the process highly attractive for expansion to an industrial scale especially considering that both the chemical and physical characterizations of our leucite product are highly satisfactory. Last but not least we explain some inferences that can be obtained from this process of synthesis in order to a better understanding of some natural occurrences of leucite in geologic systems related to basaltic magmas.

## Introduction

Leucite is a potassium aluminium silicate mineral that has become a very important component in Porcelain-Fused-to-Metal (PFM) and all ceramic restoration systems. Kelly *et al*.^1^ illustrate that leucite has the potential to reproduce the translucency, depth of colour, and texture of natural teeth. Mackert *et al*.^2^ explain that the high strength and high coefficient of thermal expansion (CTE) of leucite can improve the CTE of PFM systems to resemble that of metal substrate. Moreover, as its refractive index is similar to the glass phase in dental porcelain, leucite will enable dental ceramics to allow regular and diffuse transmission, as well as diffuse and specular reflectance of light^3^. Additionally, leucite occurs in natural systems, mainly as phenocrysts in rare high-K alkaline volcanic rocks in some restricted geodynamic contexts (i.e. in leucitic phonolites of the Roman and the Campanian volcanic provinces in Italy^4^, and references therein) and as a minero-petrological oddity in xenoliths of volcanic rocks (i.e. in the Vesuvius pyroclastics^5,6^).

Synthesis of leucite has been achieved in the past by various methods, including the solid state method^7–11^, the sol-gel method^12–14^, the molten salt technique;^8,15^ the coprecipitation method^16^, the hydrothermal cation exchange from analcime^17,18^ and the technique from a hydrothermally-derived precursor^3,17,19,20^.

Regarding the last method, Novotna *et al*.^17^ report the hydrothermal synthesis of an amorphous precursor (2 h, 200 °C) and its subsequent high temperature treatment at 1000 °C with formation of leucite. Also Zhang *et al*.^3^ report leucite synthesis obtained from a hydrothermally derived precursor and explain that the hydrothermal method is the most economical and convenient to prepare pure materials with fine particle size at low temperatures.

Kohoutkova *et al*.^19^ developed a preparation procedure of leucite from amorphous precursors; leucite precursors were prepared by a hydrothermal route at the temperatures of 100°, 150°, and 200 °C (from 1 to 72 h) and their successive calcination was performed at 1000 °C. Regarding starting materials used in conjunction with the hydrothermal method, various silica sources have also been tested. Zhang *et al*.^3^, for example, used silica sol; Kohoutkova *et al*.^16^ tested various materials such as colloidal SiO_2_ and fumed silica while attaining the most favourable outcome using an amorphous SiO_2_ powder.

In recent years, rising costs associated with synthetic processes and chemical reagents have compelled researchers to opt for alternative materials originating from natural resources. Our research group has been testing for years abundantly available georesources for use in mineralogical synthesis processes that may also prove economically beneficial at the industrial level^21–27^. The aim of the present work is to evaluate a hydrothermal preparation procedure of leucite from an amorphous precursor by testing a new starting material as silica source, *i*.*e*. a naturally derived amorphous silica from a diatomitic rock (Crotone, southern Italy). In particular, the present work assesses the value of a material obtained from an abandoned quarry, as well as aiming towards achieving both cost effective and “greener” paths for mineralogical synthesis processes. This natural, inexpensive and abundant geologic resource has been used in synthesis processes in the past, especially in the preparation of zeolitic minerals and wollastonite^21–24^ with an inferable economical advantage at larger scales. A natural, cost effective starting material can make this experimental route especially attractive when expanded to an industrial scale as long as the material properties of the leucite product remain satisfactory.

## Materials and Methods

**Figure 1:**
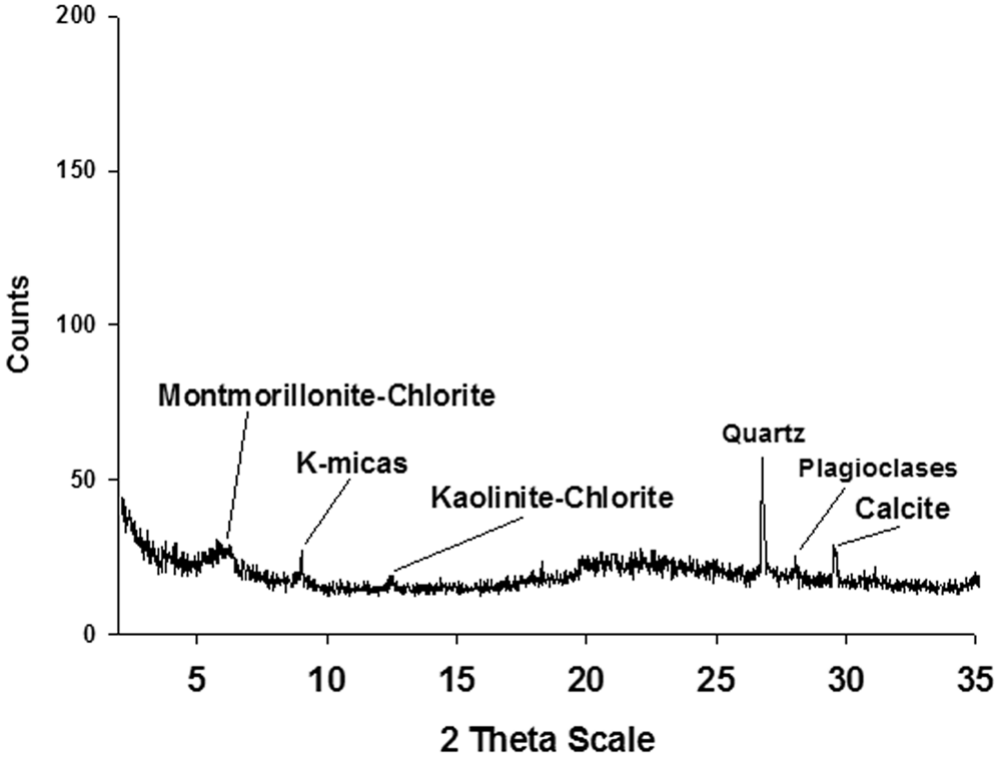
XRPD pattern of “Tripoli rock”.

The starting material was a diatomite, *i*.*e*. a tripolaceous siliceous rock (Tripoli rock) cropping out in the Crotone Basin in southern Italy. Tripoli was analysed by X-ray diffraction (XRPD) with a Siemens D5000 operating with a Bragg-Brentano geometry; CuKα = 1.518 Å, 40 kV, 40 mA, 2–35° scanning interval, step size 0.020° 2θ with a scan rate of 13 sec/step. Characterization of this material revealed a mineralogical assemblage mainly consisting of an amorphous siliceous fraction (diatoms and sponges, visible as the bulge in the range 17–25°2theta) with minor presences of quartz, montmorillonite, chlorite, kaolinite, K-micas and small amounts of calcite (Fig. 1). Chemical analysis of Tripoli rock is reported in Table 1 and was performed by X-ray fluorescence analysis (Axios-Max Advanced Panalytical; 60KV; 160 mA; 4000 W; 0.0001°2 θ). We considered the value of LOI of 1 g of sample obtained in ceramic meltpots running in an oxidizing furnace.

**Table 1:** Chemical composition of “Tripoli rock” analysed by X-ray fluorescence.

	Tripoli rock
SiO_2_	81.07 (0.45)
TiO_2_	0.26 (0.02)
Al_2_O_3_	5.03 (0.02)
Fe_2_O_3_	2.14 (0.03)
MnO	0.07 (0.01)
MgO	1.11 (0.01)
CaO	1.72 (0.03)
Na_2_O	0.25 (0.02)
K_2_O	0.68 (0.02)
P_2_O_5_	0.07 (0.01)
LOI	7.73 (0.03)
Tot.	100.13 (0.18)

The standard deviation values calculated for three analyses are reported in brackets. LOI: loss on ignition.

The synthesis of leucite was conducted through the mixing of silicate and aluminate solutions. These solutions were prepared according to the procedure already described in Novembre *et al*.^24^ In the present study, 5.19 g of the ground and powdered Tripoli material were treated with HNO_3_ (65%), in order to dissolve the calcite fraction in order to remove the soluble calcite fraction from the starting material. The diatomitic sample (ca. 5 g after the HNO_3_ treatment) was added to 50 mL of KOH (6.8%). This solution was thoroughly mixed with a magnetic stirrer for 2 h and then put in a teflon reactor/bomb and heated in an oven at 80 °C for 24 h. After filtration, the remnant solid and insoluble fraction, which consisted of clay minerals and quartz, was separated from the silicate solution. The resulting molar composition of the solution was 0.060 K_2_O–0.026SiO_2_–0.625H_2_O with traces as follows: 2.01 ppm Mg, 2.11 ppm Ca and Al, Ti and Mn lower than 0.1 ppm. Based on a mass balance calculation following this step-wise chemical separation process, the Tripoli rock was determined to be composed of: 63.27 wt% amorphous silica (diatoms and sponges), 32 wt% of clay minerals and quartz, and 3.83 wt% calcite.

The aluminate solution was prepared as follows: 0.45 g of Al(OH)_3_ (65%) was mixed with 50 mL of KOH (6.8%). The obtained aluminous solution with a composition of 0.060 K_2_O–0.0076Al_2_O_3_–0.625 H_2_O (Mn, Ti and Mg < 0.01 ppm; Fe < 0.4 ppm; K, Ca and Si < 0.2 ppm) was then heated at 100 °C for one hour.

A series of three syntheses were carried out by varying the volume ratio of the two solutions according to Table 2.

**Table 2:** Starting mixture and relative obtained mineralogical assemblages of experimental runs.

synthesis run	starting mixture	SiO_2_/Al_2_O_3_	mineralogical assemblage
1	10 ml siliceous sol + 10 ml aluminous sol	3.40	KAlSi_2_O_6_ + KAlSIO_4_-O1 (1.5–20 h); KAlSi_2_O_6_ (24 h)
2	12.5 ml siliceous sol + 7.5 ml aluminous sol	5.70	KAlSi_2_O_6_ + KAlSIO_4_-O1 (1.5–15 h); KAlSi_2_O_6_ (20 h)
3	10 ml siliceous sol + 5 ml aluminous sol	6.80	KAlSi_2_O_6_ + KAlSIO_4_-O1 (3 h); KAlSi_2_O_6_ (15–20 h)

**Figure 2:**
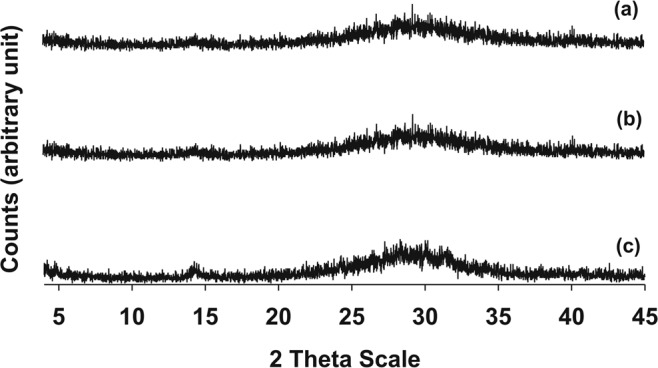
XRPD patterns of the hydrothermal gel precursors. (a): synthesis run 1; (b): synthesis run 2; (c) synthesis run 3.

The reactants were vigorously mixed for two hours with a magnetic stirrer. Each mixture was heated inside an autoclave at 150 °C and ambient pressure for a duration of one hour. The hydrothermally derived gel precursors were recovered from the reactors, filtered from the solution, thoroughly washed with distilled water and dried in an oven at 40 °C for 24 hours. These gel products were examined by XRPD analysis (Fig. 2) in order to assess their amorphous character. The three gel precursors were then calcined at 1000 °C with periodic sampling carried out at scheduled intervals.

All intermediate and final products of the three syntheses were analysed by XRPD under the same operating conditions as those for the “Tripoli rock” analysis. Identification of phases and relative peak assignment were made with reference to the following JCPDS codes: 00-038-1423 for leucite and 00-011-0579 for KAlSIO_4_-O1. The amounts of both the crystalline and amorphous phases in the synthesis powders were estimated using Quantitative Phase Analysis (QPA) applying the combined Rietveld and Reference Intensity Ratio (RIR) methods; corundum NIST 676a was added to each sample, amounting to 10%, and the powder mixtures were homogenized by hand-grinding in an agate mortar. Data for the QPA refinement were collected in the angular range 5–70° 2θ with steps of 0.02° 2θ and 10 s step^−1^, a divergence slit of 0.5° and a receiving slit of 0.1 mm.

Data were processed with the GSAS software^28^ and the graphical interface EXPGUI^29^. The unit cell parameters were determined, starting with the structural models proposed by Dove *et al*.^30^ for leucite and Kremenovic *et al*.^31^ for KAlSiO_4_-O1. Parameters were refined following Novembre *et al*.^25^.

Analysis of synthesized powders was performed by inductively coupled plasma optical emission spectroscopy (ICP-OES, Perkin Elmer Optima 3200 RL) after alkaline fusion of the sample in a Pt crucible (lithium meta-tetra borate pearls, at 3/2 ratio) and subsequent acid solubilization^27^.

Scanning electron microscope (SEM) analyses were carried out with a JEOL JSM-840 with operating conditions of 15 kV and window conditions ranging from 18 to 22 mm, following the procedure as explained in Ruggieri *et al*.^32^.

Vibrational spectra of the synthesized products were obtained with an Infrared spectrometer FTLA2000, equipped with SiC (Globar) filament source, KBr beamsplitter and DTGS detector. Samples were prepared according to the method of Robert *et al*.^33^ using powder pressed pellets (sample/KBr ratio of 1:100); spectra were processed with the program GRAMS-Al.

Thermal behaviour of gel precursors were studied by differential thermal analysis and thermogravimetry (DTA-TG) by means of a Mettler TGA/SDTA851^e^ instrument (10°/minute from 30° to 1100 °C, using an approximate sample weight of 10 mg in Al_2_O_3_ crucible).

Density of leucite was measured by He-picnometry using an AccuPyc 1330 pycnometer.

## Results

**Figure 3:**
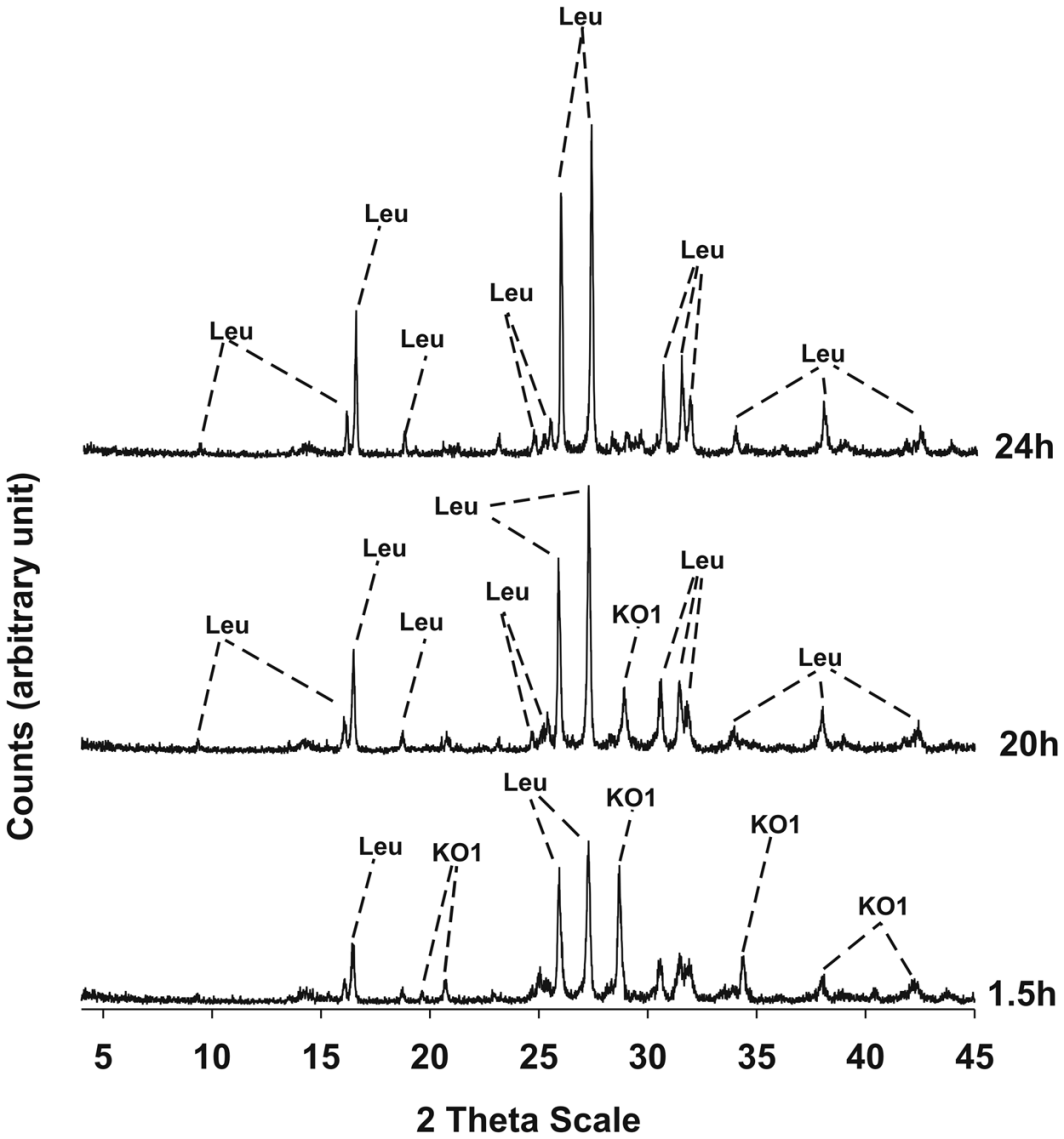
X-ray diffractometric sequence of the products of the synthesis run 1. Leu: leucite; KO1: KAlSiO_4_-O1.

**Figure 4:**
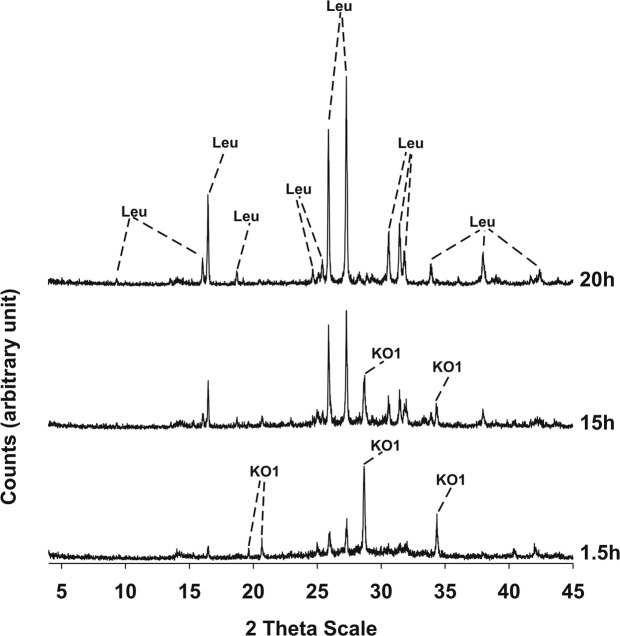
X-ray diffractometric sequence of the products of the synthesis run 2. Leu: leucite; KO1: KAlSiO_4_-O1.

**Figure 5:**
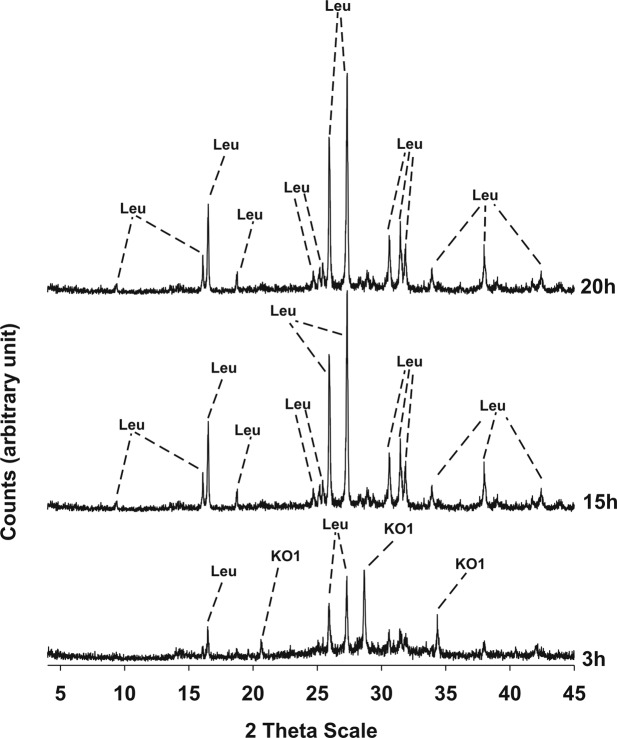
X-ray diffractometric sequence of the products of the synthesis run 3. Leu: leucite; KO1: KAlSiO_4_-O1

Results of XRPD analyses conducted on the three synthesis runs are illustrated in Figs 3–5 and the mineralogical assemblages for each run synthesis are reported in Table 2. In synthesis run 1 the crystallization of synthetic leucite is associated to that of KAlSiO_4_-O1 in the time interval 1.5–20 h; at 24 h disappearance of KAlSiO_4_-O1 is observed as it becomes replaced by leucite. Also in synthesis run 2 the crystallization of leucite is associated with KAlSiO_4_-O1 as it is shown in the diffraction patterns of the products over the time interval 1.5–15 h. After 20 h, the synthesis product consists only of leucite. In synthesis run 3 the presence of leucite already overlaps with KAlSiO_4_-O1 at 3 h; after this KAlSiO_4_-O1 becomes unstable and leucite is the lone phase in the time interval 15–20 h.

From an analysis of the above observations, it is clear that all three run syntheses result in the eventual crystallization of leucite. However, the presence of KAlSiO_4_-O1 in the XRD pattern from the earliest sampling is observed in each of the three cases indicating the compatibility of this phase under these operative temperatures^24^. Other authors have also reported the presence of a metastable phase during crystallization of leucite. Zhang *et al*.^3^ for example, found that kalsilite crystallizes as the common metastable intergrowth during leucite crystallization process. These authors carried out thermal treatments of their precursor at temperatures of 750°, 800°, 850° and 900 °C always revealing the presence of metastable kalsilite during leucite crystallization; in particular, leucite became the sole crystalline phase and kalsilite disappeared completely only at 900 °C.

**Figure 6:**
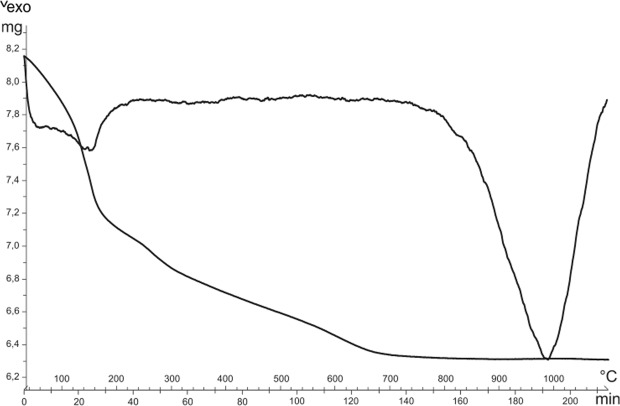
Thermo-gravimetric and differential analyses of the hydrothermal gel precursor of the synthesis run 3

In the present work KAlSiO_4_-O1 consistently crystallizes at first, then reacts with redundant silica to form the more stable leucite phase. In particular, run synthesis 3 seems to be superior from a kinetic point of view, as disappearance of metastable KAlSiO_4_-O1 is complete and an isolated pure leucite phase is reached at only 15 h. For this reason, synthesis run 3 was considered for additional characterizations. The DTA-TG curve conducted on the gel precursor of synthesis run 3 revealed a gradual and continuous water loss up to 700 °C (Fig. 6) and attributed to water evaporation, decomposition of structure water, as well as fusion of the glass. An exothermic peak is evidenced at 985 °C which is attributed to the formation of a crystalline phase. Considering the fully amorphous character of the precursor and the observed metastability of KAlSiO_4_-O1 in the synthesis of leucite, it can be assumed that the exothermic peak located at 985 °C is attributable to the formation of KAlSiO_4_-O1.

**Figure 7:**
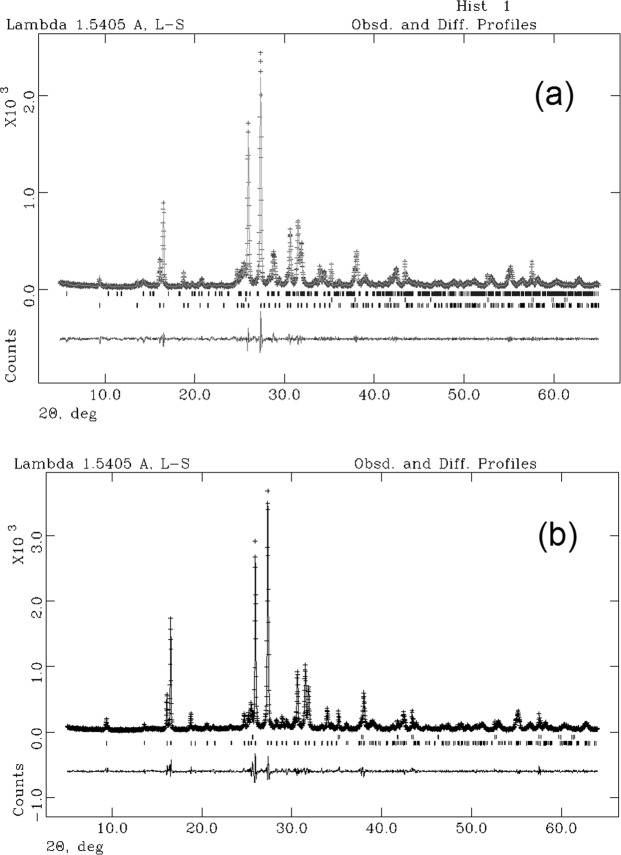
Rietveld refinement plots for synthesis run 3: experimental data (+), calculated values (—), and difference (lower trace). (**a**) Leucite + KAlSiO_4_-O1 + 10 wt % corundum NIST 676a (3h) and (**b**) Leucite + 10 wt % corundum NIST 676a (15 h). Tick marks represent the positions of the Bragg peaks. From the bottom: Leucite, corundum and KAlSiO_4_-O1 (**a**); Leucite, corundum (**b**).

In Fig. 7a the observed and calculated profiles and difference plot for leucite, KAlSiO_4_-O1 and corundum NIST 676a with tick marks at the positions of the Bragg peaks are reported for the samples at 3 h. The cell parameters of leucite, refined with tetragonal symmetry, space group *I*4_1_/*a*, are *a*_0_ = *b*_0_ = 13.07 *Å* and *c*_0_ = 13.75 *Å*; the cell parameters of KAlSiO_4_-O1, refined with monoclinic symmetry, space group *P2*_1_, are *a*_0_ = 15.62, *b*_0_ = 9.05 *Å* and *c*_0_ = 8.56 *Å* (Table 3). The results of the QPA analysis indicate that the calculated amorphous phase in the sample at 3 h is 5.4%, thus resulting in a final crystalline product of 15.95% KAlSiO_4_-O1 and 78.65% leucite.

**Table 3:** Experimental conditions and crystallographic data for Leucite and KAlSiO_4_-O1 plus corundum Nist 676a: samples at 3 and 15 h of synthesis run 3.

sample	3 h, synthesis run 3 + 10% Corundum NIST 676a	15 h, synthesis run 3 + 10% Corundum NIST 676a
Wavelenght (Å)	1.5418	1.5418
No. of observation	2248	1589
*R*_*wp*_	0.13	0.14
*R*_*p*_	0.09	0.11
*CHI* ^2^	1.51	2.18
% amorphous	5.40(11)	0.11(8)
% phase KAlSiO_4_-O1	15.95(14)	0
% phase leucite	78.65(15)	99.89(18)
Space group leucite	*I*4_1_/*a*	*I*4_1_/*a*
*a (Å)*	13.0712(51)	13.0614(18)
*b (Å)*	13.0715(16)	13.0671(38)
*c (Å)*	13.7511(17)	13.7544(16)
Space group KAlSiO_4_-O1	P2_1_	/
*a (Å)*	15.6223(21)	/
*b (Å)*	9.0518(8)	/
*c (Å)*	8.5641(9)	/

In Fig. 7b the observed and calculated profiles and difference plot for leucite and corundum NIST 676a with tick marks at the positions of the Bragg peaks are reported for the samples at 15 h. The cell parameters of leucite are *a*_0_ = *b*_0_ = 13.06 *Å* and *c*_0_ = 13.75 *Å* (Table 2). The results of the QPA analysis indicate that the calculated amorphous phase in the sample at 15 h is 0.1%, thus resulting in a final product of 99.9% leucite.

**Figure 8:**
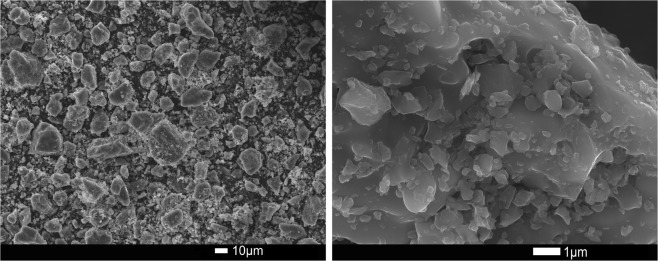
SEM image of a sample synthesized at 15 h of run synthesis 3.

Figure 8 shows SEM micrographs of the sample from run synthesis 3 at 15 h. Differently shaped particles of size around 10 micron can be observed. This fine leucite grains can improve characteristics such as reliability, flexural strength of human teeth.

In Table 4 we report the chemical analysis of the synthesized leucite (sample at 15 h). Values are coherent with those reported for this mineral by Klouzkova *et al*.^34^. The chemical formula was calculated based from Table 4 to be K _15,64_ Al _16,12_ Si _31,99_ O _96_.

**Table 4:** Chemical characterization of the product of synthesis run 3.

sample	K_2_O (%)	SiO_2_(%)	Al_2_O_3_ (%)	Time (h)	XRD spectrum
ICP -6	21.16	55.20	23.63	15 h	leucite (strong)

Density value of 350.9 Kg m^−3^ was calculated for leucite, which is comparable to that obtained by Balandis and Sinkyavichene^18^.

**Figure 9:**
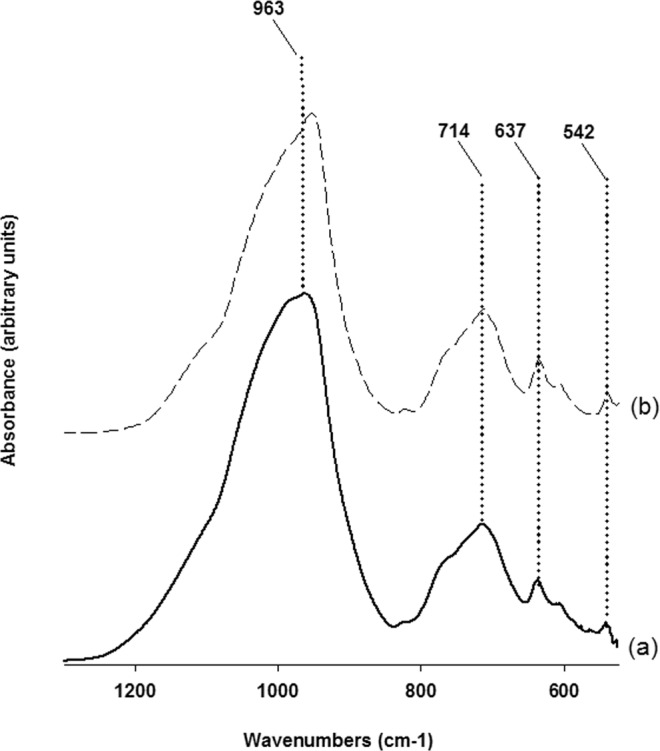
Infrared spectra of the sample at 15 h of synthesis run 3.

Infrared spectra obtained from sample at 15 h of synthesis run 3 is shown in Fig. 9a. The spectrum consists of a strong band covering a broad region (1250–830 cm^−1^) characteristic of T-O (T = tetrahedral cation) stretching vibrations with its maximum located at 963 cm^−1^. In the region of polyhedral deformation (800–400 cm^−1^) three bands are evidenced and positioned respectively at 714, 637 and 542 cm^−1^. These band positions are in good agreement with those of a hydrothermally synthesized leucite from Balandis and Sinkyavichene^18^. Figure 9b is the infrared absorption spectrum of a leucite from Roccamonfina (Italy) and shows near perfect correspondence to that of our sample spectrum with only a small displacement of the major peak.

## Discussion and Conclusion

The principle aim of our study was to synthesize leucite using a natural, inexpensive and abundant geologic resource through a hydrothermal procedure. We chose to prepare the silicate solution using a diatomitic rock (Crotone, southern Italy) containing more than 60 wt% SiO_2_. Three mixtures of varying ratios of aluminate and silicate solutions were prepared and subjected to hydrothermal treatment at 150 °C for one hour. It is evident from Table 2 that these different mixtures were necessary as trials due to the nature of the gel producing reaction necessitating an initial Si:Al ratio greater than the stoichiometric ratio of 2:1 in leucite. Subsequently all three of the hydrothermal precursors were subjected to calcination at a temperature of 1000 °C. Synthesis run 3 was considered to be the more efficient trial from a kinetics standpoint as the reaction to form leucite was completed in 5 fewer hours compared to synthesis run 2 and 9 fewer hours than synthesis run 1. Interestingly, the nominal Si:Al ratio of the original solution mixture for this run had the greatest deviation from leucite stoichiometry. This kinetic aspect becomes very important regarding any possible industrial application of the synthesis protocol, as a means of minimizing total production costs by reducing synthesis times.

The chemical-physical and spectroscopic characterization of the experimental products substantiate the efficiency of the experimental protocol adopted as we can compare our results to those from earlier studies. Kohoutkova *et al*.^19^ for example, also synthesized leucite starting from an amorphous precursor obtained by the thermal treatment of silicate and aluminate solutions at 150 °C and subsequently subjected to calcination at 1000 °C. However, the authors did not perform any systematic sampling of the synthetic powders during calcination and the duration of their synthesis was limited to 4 h. Neither chemical nor physical characterization of their leucite product was provided thereby making them unable to rule out the possible presence of amorphous material due to incomplete crystallization after 4 h. In our case, the periodic samplings conducted during the calcination treatment have made it possible to better define the kinetic impediments to this reaction under the conditions of our synthesis.

The use of amorphous silica obtained from a georesource, rather than of commercial origin, increases the attractiveness of our protocol by way of its considerably lower cost without compromising the end product. A substantial difference between our work and those of others also lies in the effective assessment of the degree of success of the experiment in quantitative terms through QPA of the percentage of crystallization *vs*. amorphous material. Industrial methods require a minimum efficiency level of 90% in order to operate. Progressive sampling over time, in fact, allows for a tunable efficiency such that specific calcination times can be imposed to meet targeted levels of phase purity. Moreover, the results of the QPA analysis, which indicates a final product consisting of >99% leucite suggests that transfer to an industrial production scale would be very feasible.

The studied synthetic route does not allow us to infer any direct interpretation on crystallization of leucite phenocrysts in phonolitic rocks, since a number of hypotheses clearly have associated magmas that generate these rocks to phenomena of crustal assimilation (see^4^ and references therein). On the other hand, this type of synthesis can be applied in better understanding the genesis of leucite crystals in geodic-like cavities of limestone xenoliths in Vesuvius rocks^5,6^, and even as irregular, large patches in mesostase of basanitic or basaltic (i.e. in NE Spain^35^). In natural systems an association of K and free Al and Si is required in order to generate leucite by pyrometamorphism or by a succession of hydrothermal and pyrometamorphic processes. Non crystalline forms of silica are not uncommon in nature (i.e. diatomite like that used here in our synthesis experiments; and a number of other sedimentary or volcanic glassy materials). These clays eventually could be also an additional source of potassium. Metakaolin is not uncommon in thermal metamorphic environments, or in pyrometamorphic ones (i.e. the origined by autocombustion of outcropping coal seals). A relatively reduced number of K sources might be envisaged: K-feldspar is a very improbable one, since the range of temperatures in order to reach breakdown of the silicate framework is too high for most of the eventual magmas potentially involved. Other eventual K sources are: K-rich volcanic glass or tonstein deposits, evaporitic deposits (i.e. silvite-carnalite paragenesis), and organic-rich sediments or wood fragments. Ash from caducifolia wood is a well-know source of potassium carbonate since medieval times (i.e. in the glass production process of forest glass, see^36^ and references therein), and recent experiments^37^ show that combustion of wood and other vegetal matter can lead to vaporization for most of their K content. In the same way hydrothermal conditions (similar to the ones in the first step of our experiment, and comparable to the ones in natural hydrothermal to pyrometamorphic transient conditions associated with shallow intrusion of basaltic magmas, see^38^ and references therein) are associated with free silica and can provide the retention of K as a reactant in the crystallization system. Therefore, the synthesis here described can be useful in the interpretation of natural systems characterized by transient hydrothermal to pyrometamorphic conditions involving glass, tonstein or clay-rich sedimentary xenoliths and organic matter (i.e. wood) associated to shallow basaltic intrusions.
